# Identifying functional populations among the interneurons in laminae I-III of the spinal dorsal horn

**DOI:** 10.1177/1744806917693003

**Published:** 2017-02-01

**Authors:** Andrew J Todd

**Affiliations:** Institute of Neuroscience and Psychology, College of Medical, Veterinary and Life Sciences, University of Glasgow, Glasgow, UK

**Keywords:** Itch, neuropeptide, pain, spinal cord

## Abstract

The spinal dorsal horn receives input from primary afferent axons, which terminate in a modality-specific fashion in different laminae. The incoming somatosensory information is processed through complex synaptic circuits involving excitatory and inhibitory interneurons, before being transmitted to the brain via projection neurons for conscious perception. The dorsal horn is important, firstly because changes in this region contribute to chronic pain states, and secondly because it contains potential targets for the development of new treatments for pain. However, at present, we have only a limited understanding of the neuronal circuitry within this region, and this is largely because of the difficulty in defining functional populations among the excitatory and inhibitory interneurons. The recent discovery of specific neurochemically defined interneuron populations, together with the development of molecular genetic techniques for altering neuronal function *in vivo*, are resulting in a dramatic improvement in our understanding of somatosensory processing at the spinal level.

## Basic organisation of the dorsal horn

The dorsal horn of the spinal cord is the main site of termination for primary somatosensory afferent axons, and contains the first synapses in neuronal pathways that transmit sensory information that is perceived as pain and itch.^1–3^ In 1965, Melzack and Wall proposed that this region, and in particular the substantia gelatinosa (lamina II), played a key role in modulating somatosensation, by selectively inhibiting primary afferent inputs before these were transmitted to the brain through ascending pathways.^4^ It is now known that the neuronal organisation and synaptic circuitry of the region are far more complex than could have been imagined at the time of Melzack and Wall's Gate Theory. However, the basic assumption that the superficial dorsal horn modulates nociceptive input is now universally accepted. This has led to numerous studies that have investigated the mechanisms underlying this phenomenon, with a view to identifying novel targets for the treatment of pathological pain states. A major goal has been to define the synaptic circuits involving the various neuronal components of the dorsal horn. In addition to terminal arbors of primary afferents, the dorsal horn contains descending axons from various brain regions, together with a large number of neurons, which include both projection cells and interneurons.

Primary afferents have been assigned to functional classes based on their conduction velocity and their responses to natural stimuli, together with a variety of neurochemical features.^3,5^ Several major classes of nociceptor have long been recognised, including myelinated (predominantly Aδ) afferents that terminate in lamina I^6^ and two broad classes of unmyelinated afferents: those that contain neuropeptides such as substance P and calcitonin gene-related peptide and arborise mainly in lamina I and the outer part of lamina II (IIo), and a non-peptidergic class that bind the lectin IB4, express the mas-related G protein-coupled receptor MrgD (C^MrgD^ afferents) and terminate in mid-lamina II.^7^ These are thought to be involved in different aspects of pain, since ablation of the peptidergic afferents results in loss of thermal pain, while the MrgD population is required for the full expression of mechanical pain.^8^ Low-threshold mechanoreceptors include unmyelinated (C-LTMR) and myelinated (A-LTMR) types, and can be classified by axon diameter and receptive field properties, with the different types having characteristic zones of termination that extend from the inner part of lamina II (IIi) to lamina V.^3,9,10^ Recent studies of dorsal root ganglion cells, involving unbiased classification through cluster analysis, suggest that there are several other distinct functional types of primary afferent that are currently less well understood.^5,11^

Dorsal horn neurons can be divided into two main types: those with axons that project to the brain (projection cells) and those with axons that remain in the spinal cord (interneurons). Several different classes of projection cell can be recognised based on the supraspinal target(s) of their axons,^3^ but those most relevant to pain and itch perception belong to the anterolateral tract (ALT). ALT projection neurons are most densely packed in lamina I and are scattered throughout the deeper dorsal horn laminae (III-VI). Their postsynaptic targets, which are mainly contralateral, include the thalamus, periaqueductal grey matter, lateral parabrachial area, and various parts of the medullary reticular formation, with many ALT cells sending axon collaterals to several of these regions and some projecting bilaterally.^12–14^ These cells are glutamatergic,^15^ and since some of them give rise to collateral axons in the spinal cord,^16^ they also contribute to local excitatory synaptic circuits.

The great majority of the neurons in laminae I-III are interneurons. These account for virtually all of the neurons in lamina II and 90%–95% of those in lamina I.^17,18^ It is more difficult to estimate the proportion in lamina III, because this region contains projection cells belonging to several different tracts.^3^ However, it is likely that most neurons in this lamina are also interneurons. Although the interneurons in laminae I-III probably all give rise to local axonal arbors,^19^ it has been reported that many of them also have long propriospinal projections that can extend for several segments.^20^

The interneurons can be divided into two broad functional classes: inhibitory cells, which use GABA and/or glycine as their principal transmitter, and excitatory (glutamatergic) interneurons. These can be distinguished by using immunocytochemistry, for example, with antibodies against GABA and glycine, which are present at relatively high levels in cell bodies of the inhibitory interneurons.^21^ Virtually all of the neurons in laminae I-III that show high levels of glycine immunoreactivity are also labelled with GABA antibodies, while there are many additional cells that are only GABA-immunoreactive. This suggests that most of the inhibitory interneurons in this region release GABA, and that some of these cells also release glycine. However, whole-cell recordings from neurons in the superficial laminae show that these frequently have inhibitory postsynaptic currents that are mediated by glycine and not GABA,^22,23^ and it has been suggested that this results from differential distribution of GABA_A_ and glycine receptors at inhibitory synapses.^23^ Although inhibitory interneurons are uniformly distributed throughout laminae I-III, those enriched with glycine are particularly numerous in lamina III.^21^ GABAergic and glycinergic neurons can also be revealed by their expression of the GABA-synthesising enzyme glutamate decarboxylase (GAD) and the neuronal glycine transporter GlyT2, respectively, and these show a laminar distribution consistent with that described above.^24^ We have recently reported that in the mouse, inhibitory interneurons account for 26% of the neurons in laminae I-II and 38% of those in lamina III.^25^ It is likely that all of the remaining neurons are glutamatergic,^19^ and these would include both excitatory interneurons and projection cells. Immunostaining for the amino acid transmitters is technically difficult, and a more convenient indirect approach is use antibodies against transcription factors that define inhibitory or excitatory phenotypes: Pax2 and Lmx1b, respectively.^26^ We have found that the proportions of neurons that are Pax2-immunoreactive in laminae I-II and in lamina III are very close to the proportions that show GABA and/or glycine immunoreactivity,^25,27^ which is consistent with the suggestion that Pax2 is a reliable marker for the inhibitory interneurons in the adult spinal cord.^28,29^

Early studies in which GABA_A_ or glycine receptor antagonists were applied to the spinal cord by intrathecal injection showed that this resulted in exaggerated pain responses.^30,31^ Although some GABAergic/glycinergic synapses in the superficial dorsal horn involve axons that originate from the brainstem,^32^ it is likely that the great majority are formed by the axons of local inhibitory interneurons. These findings therefore provided evidence that an important role of the inhibitory interneurons was to maintain an appropriate level of pain (or lack of pain) in response to a peripheral stimulus.^31^ Until recently, much less has been known about the functions of the excitatory interneurons, although it is becoming apparent that they have an important role in both acute and pathological pain.^33,34^

## Classification of the interneurons

It is clear that both the excitatory and the inhibitory interneurons consist of several different functional populations, and there have therefore been many attempts to define these populations. Elsewhere in the central nervous system (CNS), neuronal morphology has provided a valuable way of recognising functional populations of interneurons,^35^ and a similar approach has been applied to the superficial dorsal horn. This was initially achieved by using Golgi impregnation to provide sparse labelling of apparently random types of neuron.^36,37^ More recently, neuronal morphology has been studied following whole-cell patch-clamp recording from spinal cord slices in vitro.^19,38–50^ A major advantage of the latter approach is that it allows correlation of morphology with electrophysiological properties, for example, action potential firing pattern in response to injected depolarising current.^38,41,44,48,50,51^ In addition, it is possible to compare morphology with neurotransmitter phenotype, which can be determined by staining for specific vesicular transporters in the axons of recorded neurons^19^ or by the use of mouse lines in which either inhibitory or excitatory interneurons are selectively labelled with a fluorescent protein.^44,50^

Although both morphological and electrophysiological properties can be used to classify some of the interneurons in this region (see below), neither has provided a comprehensive classification scheme.^2^ The superficial dorsal horn contains an extensive array of neurochemical markers, including neuropeptides and their receptors, calcium-binding proteins and a variety of enzymes. These frequently show a specific laminar pattern, and are often differentially distributed among excitatory and inhibitory interneurons.^52^ These findings have allowed us to define several neurochemically distinct populations among both of these broad classes.^53,54^ In addition, recent studies have used the transient developmental expression of certain proteins to identify interneuron populations in the dorsal horn.^34,55^ A major advantage of using neurochemistry to define neuronal populations is that these can be targeted for electrophysiological recording to provide detailed functional information about the neurons.^33,34,44,50,55–66^ In addition, it allows manipulation of their function through molecular genetic techniques in which a recombinase drives expression of another protein that can be used to activate, inhibit, silence or ablate the neurons under investigation.^28,33,34,55,61,67,68^ Neuronal populations that can be defined by expression of neuropeptide receptors have also been targeted by intrathecal administration of the ribosome-inactivating toxin saporin conjugated to the corresponding neuropeptide.^69,70^ Since all of these methods can be applied in vivo, they can reveal the effects of activating or inactivating different interneuron populations on responses to painful or pruritic stimuli.

## Populations defined by morphological and electrophysiological properties

**Figure 1.**
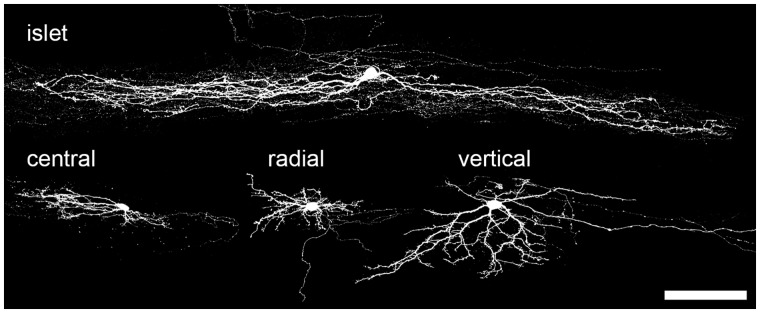
Confocal images of four lamina II neurons recorded in parasagittal spinal cord slices from young adult rats in the study by Yasaka et al.^19^ Neurobiotin in the pipette allowed labelling with fluorescent avidin after whole-cell recording. The cells correspond to the four main classes recognised by Grudt and Perl.^38^ Reproduced with permission from Yasaka et al.^19^

The most widely used morphological scheme for classifying interneurons in lamina II is that developed by Grudt and Perl,^38^ who identified four major types: (1) islet cells, which have dendritic arbors that are highly elongated along the rostrocaudal axis; (2) vertical cells, which are generally located in the outer half of lamina II (lamina IIo) and typically have dendrites that extend ventrally from the soma within a cone-shaped volume; (3) radial cells, which have relatively short dendrites that extend along both rostrocaudal and dorsoventral axes; and (4) central cells, which resemble islet cells, but have much smaller dendritic trees (Figure 1). Certain patterns of axonal arborisation were noted for each class: vertical cell axons often extended into lamina I, and it was subsequently shown that these could innervate ALT projection cells in this lamina.^46^ In contrast, the axons of islet, radial and central cells were generally centred on lamina II, with some extension into the adjacent laminae. There were also differences in the firing patterns of the different classes: islet cells and some of the vertical cells fired tonically when injected with depolarising current, while the remaining vertical cells and the radial cells showed a delay before the first action potential. The central cells were further divided into three subtypes, based on their firing pattern in response to injected depolarising current: tonic and transient firing. The transient firing cells were further subdivided into those with or without an A-type potassium (*I*_A_) current. It has also been shown that cells belonging to these four classes differ in their monosynaptic primary afferent inputs, in their primary afferent-evoked inhibitory inputs and in the extent to which they are interconnected in synaptic circuits.^38,40,45,46,49^ However, although the majority of neurons identified by Grudt and Perl could be assigned to one of these classes, around 25% of their sample could not be classified with this scheme.

An important question is whether cells belonging to each of these morphological classes are excitatory or inhibitory interneurons. An early study^71^ that combined Golgi staining with immunocytochemistry reported that islet cells were GABA-immunoreactive (and therefore inhibitory), while another population defined as stalked cells^37^ (which approximately correspond to Grudt and Perl's vertical class) were never GABA-immunoreactive, and were therefore assumed to be excitatory. These findings have been confirmed and extended by subsequent whole-cell patch-clamp recording studies in which the transmitter phenotype has been identified either by means of immunocytochemistry,^19,39^ by recording from cells that were genetically defined as inhibitory or excitatory^44,50^ or by recording from pairs of synaptically linked neurons and characterising the response of the postsynaptic cell.^45,46,49^ Taken together, these studies indicate that islet cells are invariably inhibitory interneurons,^19,39,44,45,49^ while the great majority of vertical cells are excitatory.^19,39,46,50^ However, a few neurons that were classified as vertical cells were found to be inhibitory, based on expression of the GABA-synthesing enzyme GAD^39,44^ or the vesicular GABA transporter (VGAT),^19^ and this indicates the need for caution in defining these cells purely on a morphological basis. Radial cells are likely to be exclusively excitatory interneurons, since they were identified among cells that express the vesicular glutamate transporter VGLUT2,^50^ but not among those expressing GAD,^44^ while their axons were found to be VGLUT2-immunoreactive.^19^ Two cells described as radial by Maxwell et al.^39^ had GAD-immunoreactive axons. However, these had much more extensive dendritic trees than the radial cells defined by Grudt and Perl.^38^ If the definition of radial cells is confined to neurons with restricted dendritic trees, these should therefore be excitatory interneurons. In the case of central cells, the situation is much less clear, and as noted above, Grudt and Perl identified three different subtypes. In addition, central cells have been identified among both the inhibitory and excitatory interneurons.^19,39,45,46,49,50,57^ It is therefore questionable whether assigning neurons to the “central” morphological class is useful, and it has been suggested that central cells, together with the remaining unclassified neurons represent morphologically heterogeneous groups of cells among both the excitatory and inhibitory interneurons.^19^

The situation is less clear for interneurons in laminae I and III. Several morphological classes have been identified among lamina I neurons,^36,41,72^ but little is known about whether cells in these classes correspond to inhibitory or excitatory interneurons, and a further complication is the presence of projection neurons in this lamina. In recent whole-cell recording studies,^73,74^ lamina I interneurons have been found among three morphological classes that were previously identified in this lamina based on Golgi staining: fusiform, flattened and multipolar cells.^36^ The flattened and fusiform cells have dendrites that are mainly restricted to lamina I, whereas multipolar cells have significant dendritic extension into lamina II and in some cases lamina III. In one of these studies, some of the recorded cells were shown to be inhibitory interneurons, based on expression of VGAT in their axonal boutons.^74^ However, these were found among both the flattened and multipolar classes, and it is therefore not clear how dendritic morphology relates to function. Lamina III also contains neurons with highly variable morphology,^42^ but again the association between morphology and neurotransmitter phenotype is poorly understood.

When neurons are injected with depolarising current, they can show several different firing patterns, such as transient (initial burst), gap, delayed and reluctant firing.^38,41,75,76^ These patterns reflect the presence of different ion channels. For example, gap, delayed and reluctant firing are thought to result from *I*_A_ current mediated by voltage-gated potassium channels, in particular K_V_4.2.^51^ There is general agreement that cells showing these A-type firing patterns are much more frequently encountered among the excitatory interneuron populations, whereas most inhibitory interneurons show tonic or transient firing.^19,44,50,57^

## Populations defined by neurochemical properties

Several neuropeptides are expressed by subsets of interneurons in laminae I-III. Some of these, for example, neuropeptide Y (NPY), galanin and nociceptin are present in inhibitory interneurons, others, such as somatostatin, neurotensin, GRP, neurokinin B (NKB), substance P and cholecystokinin are found predominantly in excitatory interneurons, while the opioid peptides enkephalin and dynorphin are expressed by both excitatory and inhibitory interneurons.^33,77–88^ A further complication is that several of these peptides (somatostatin, substance P and galanin) are normally also present at relatively high levels in primary afferents, while NPY is up-regulated in primary afferents following peripheral nerve injury. The laminar distribution of dorsal horn neurons that contain these different peptides varies considerably. For example, galanin and substance P are expressed by cells in lamina I and IIo, neurotensin and NKB by cells on either side of the lamina II/III border, somatostatin by cells throughout laminae I-II and NPY by cells throughout laminae I-III.^2,52^ This suggests that the neuropeptides may be differentially expressed by distinct functional populations of interneurons.

There are also various neuropeptide receptors in this region. Among these, the somatostatin receptor 2a (sst_2A_) is virtually restricted to inhibitory interneurons in laminae I-II and is found on around half of these cells.^25,89^ The neurokinin 1 receptor (NK1r, the receptor for substance P) is present at high levels on many projection neurons in lamina I, but is also seen on excitatory interneurons in this lamina.^15^ The NPY Y1 receptor is highly expressed in laminae I-II, where it is found mainly on excitatory interneurons.^90–92^ There has been considerable interest in the receptor for GRP (GRPR), following the finding that GRPR-expressing neurons play an important role in itch.^93,94^ These cells are restricted to laminae I-IIo, and are thought to be excitatory interneurons.^85^

Certain other proteins, including calcium-binding proteins and a variety of enzymes, are restricted to subsets of the interneurons. Among the calcium-binding proteins, parvalbumin is found in cells in the inner part of lamina II and in lamina III, the majority of which are inhibitory interneurons.^95–98^ Both calbindin and calretinin are present in many neurons throughout laminae I-II.^97–99^ Most of these are excitatory neurons, although around 12% of the calretinin cells are inhibitory.^62,95,98^ The γ isoform of protein kinase C (PKCγ) is largely restricted to a band of excitatory interneurons that occupy laminae IIi and III.^100–102^ These cells have attracted considerable attention because they are apparently necessary for the development of tactile allodynia.^100,103–105^ The neuronal form of nitric oxide synthase (nNOS) is expressed by cells that are scattered throughout the dorsal horn, with a relatively high concentration in laminae IIi-III, and most of these are inhibitory interneurons.^106–108^

**Figure 2.**
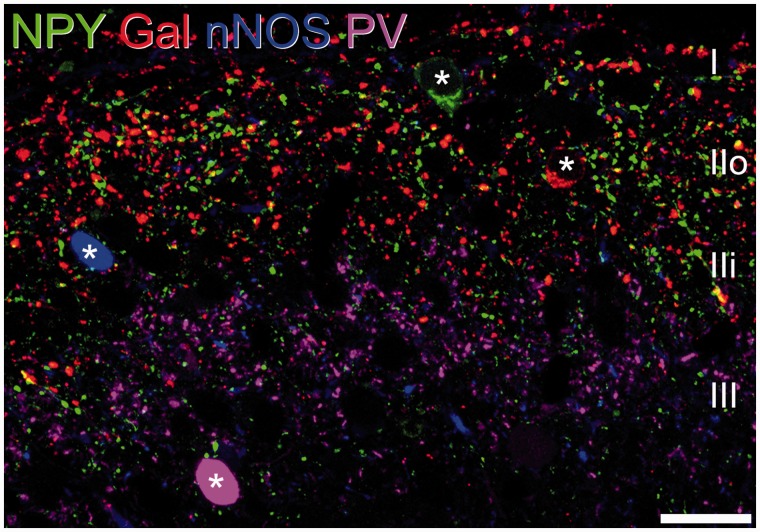
Neurochemical populations among the inhibitory interneurons. Four largely non-overlapping populations can be identified among the inhibitory interneurons in lamina I-III, defined by expression of neuropeptide Y (NPY), galanin (Gal), neuronal nitric oxide synthase (nNOS) and parvalbumin (PV). This confocal image shows a single optical plane through a transverse section of rat lumbar spinal cord that had been immunostained to reveal each of these substances. A single cell of each type is visible, and these are indicated with asterisks. Approximate positions of laminae are shown. Scale bar = 20 µm. Reproduced with permission from Battaglia AA: *An Introduction to Pain and its relation to Nervous System Disorders.* John Wiley and Sons; 2016.

It is clear from this account that there are many neurochemical markers that could be used to classify interneurons in laminae I-III. However, it is unlikely that any one of these will define a unique functional population. One strategy that has been used in an attempt to identify discrete populations has been to look for markers that are expressed in non-overlapping subsets among either the inhibitory or excitatory interneurons. For example, we have found that in laminae I-III of the rat spinal cord, four largely non-overlapping populations of inhibitory interneurons could be identified by their expression of NPY, galanin, nNOS and parvalbumin^54,109^ (Figure 2). The galanin cells largely correspond to those inhibitory interneurons that express dynorphin, although dynorphin is also present in a smaller population of excitatory interneurons that lack galanin.^81,83^ As stated above, around half of the inhibitory interneurons in laminae I-II express sst_2A_, and the sst_2A_-positive neurons include those that contain galanin/dynorphin as well as the nNOS cells. In contrast, the PV cells, and most of the NPY cells, are not sst_2A_-immunoreactive. Between them, these four populations (those expressing NPY, galanin/dynorphin, nNOS or parvalbumin) account for over half of the inhibitory interneurons in laminae I-II.^110^ We have since found a similar pattern in the mouse, although there is some co-expression of nNOS with galanin/dynorphin^29,59,111^ and we also find that some neurons contain both galanin and NPY in this species (AJT and K Boyle, unpublished data). Two further pieces of evidence link the galanin/dynorphin- and nNOS-expressing inhibitory interneurons. Firstly, they are both included among the cells that contain enhanced green fluorescent protein (eGFP) in a mouse line in which eGFP expression is under control of the prion promoter (PrP-eGFP mouse).^59^ These cells have been extensively characterised by Perl and co-workers and found to have specific synaptic connections with other neuronal populations.^49,57,112^ Secondly, we have shown that the galanin/dynorphin and nNOS populations of inhibitory interneurons are uniquely dependent on the transcription factor Bhlhb5, since they are lost in the *Bhlhb5^−^^/^^−^* mouse,^29,111^ a model of chronic itch.^113^

Various studies have examined the morphology of cells belonging to these neurochemical classes following whole-cell recording. Many of the parvalbumin cells in lamina IIi (as well as some of those in lamina III) have highly elongated dendritic trees, and can be classified as islet cells.^60,98^ However, since islet cells are found throughout lamina II,^19,38^ it is very likely that there are other populations of islet cells that lack parvalbumin. Hantman et al.^57^ reported that PrP-eGFP neurons (which include galanin/dynorphin- and nNOS-expressing inhibitory interneurons^59^) were central cells. However, we have found that they have highly variable dendritic arbors, and could not be assigned to any morphological class.^56^ NPY-expressing cells were also morphologically heterogeneous, and a cluster analysis based on dendritic morphology failed to separate NPY-expressing cells from the PrP-eGFP cells.^58^ These findings suggest that parvalbumin is expressed in a subset of islet cells, while cells that express NPY, nNOS or galanin/dynorphin cannot be classified morphologically, but are never islet cells.

**Figure 3.**
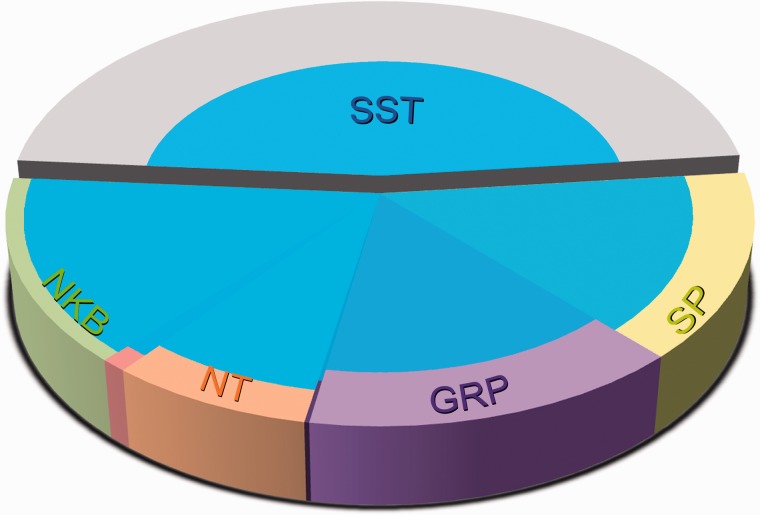
Populations of excitatory neurons in laminae I-II defined by neuropeptide expression. The pie chart shows the proportions of all excitatory neurons in this region that express neurokinin B (NKB), neurotensin (NT), gastrin-releasing peptide (GRP) or substance P (SP). The GRP cells were detected by the presence of eGFP in mice in which eGFP is under control of the GRP promoter, while the other populations were revealed by immunocytochemistry for the neuropeptide or its precursor protein. Note that there is limited overlap between the neurotensin cells and those that express NKB or GRP. Between them, these four populations account for just over half of the excitatory neurons in laminae I-II. Unlike these four neuropeptides, somatostatin is widely expressed among excitatory neurons. We have estimated that it is present in between 60% and 90% of the cells that express each of the other neuropeptides, as well as in some of the remaining excitatory neurons. For further details, see text.

**Figure 4.**
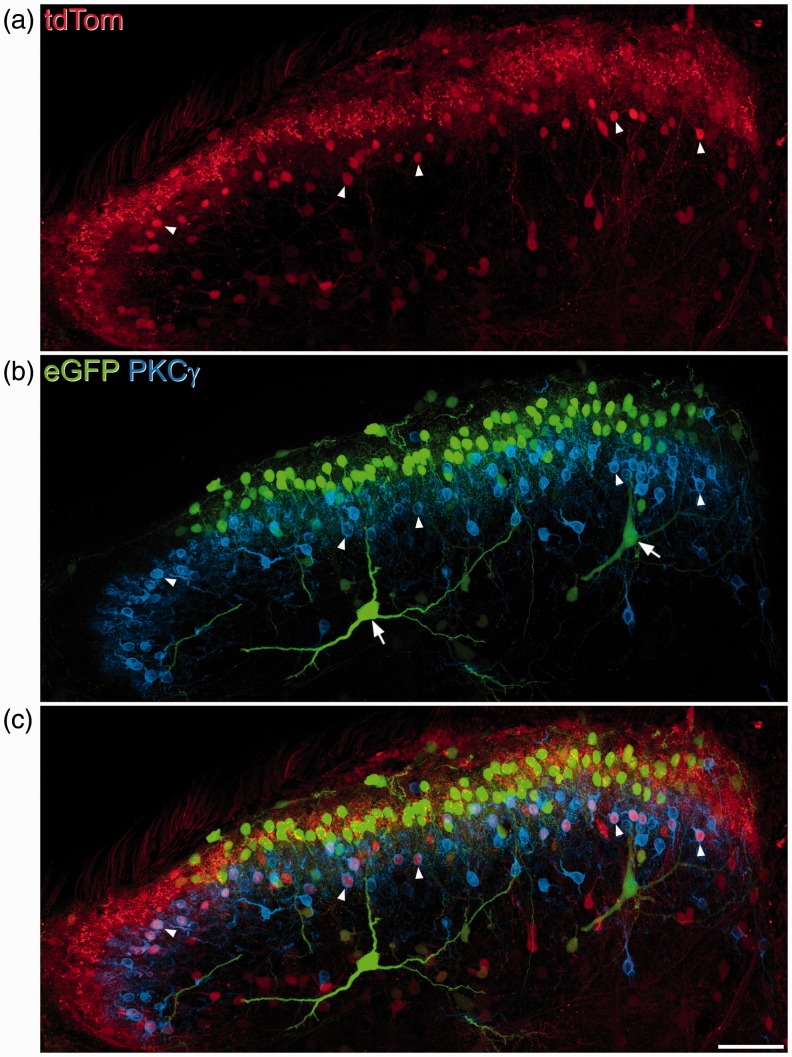
The distribution of tdTom- and eGFP-positive cells in the dorsal horn following intraspinal injection of AAV.flex.eGFP into a Tac1^Cre^;Ai9 mouse. The section has been scanned to reveal tdTom (red), eGFP (green) and PKCγ (blue). (a) TdTom^+^ neurons are concentrated in the superficial laminae and scattered through the deep dorsal horn. (b) The distribution of eGFP^+^ neurons is more restricted, as most of these lie dorsal to the band of PKCγ-immunoreactive neurons, which occupy lamina IIi. Note that none of the eGFP^+^ cells are PKCγ-immunoreactive. (c) In the merged image, it can be seen that there are many tdTom^+^ neurons that lack eGFP (and therefore appear red), and that these include PKCγ-immunoreactive cells (some indicated with arrowheads). The two large cells that are indicated with arrows are likely to be ALT projection neurons, some of which express substance P. The images are projected from 45 optical sections at 1 µm z-spacing. Scale bar = 50 µm. Reproduced with permission from Gutierrez-Mecinas et al.^27^

More recently, we have used a similar strategy to look for distinct populations among the excitatory interneurons. The calcium-binding proteins calbindin and calretinin are widely expressed by neurons in the superficial dorsal horn,^98,114^ with virtually all of the neurons that contain calbindin^95^ and around 88% of the calretinin-containing cells^62^ being glutamatergic. However, the large numbers of cells that contain each of these proteins suggest that they are not confined to specific functional populations, and it has been shown that excitatory calretinin-containing neurons include vertical, radial and central cells.^62^ Several neuropeptides are expressed mainly or exclusively by excitatory interneurons,^2,52^ and we have found that four of these peptides: substance P, GRP, neurotensin and NKB are expressed in largely separate populations in the mouse. Between them these account for just over half of the excitatory interneurons in laminae I-II^27,53^ (Figure 3), and they show distinct laminar distributions. The neurotensin and NKB populations are found in lamina IIi-III, with many corresponding to the PKCγ-expressing cells. The GRP neurons were identified in a transgenic mouse line (Tg(GRP-eGFP)) in which eGFP expression is largely restricted to cells with GRP mRNA.^115^ They occupied the central part of lamina II and showed very limited overlap with the PKCγ cells. Substance P-expressing neurons were identified with two methods: immunocytochemical detection of the substance P precursor (preprotachykinin A, PPTA) and intraspinal injection of adenoassociated virus coding for a Cre-dependent form of eGFP (AAV.flex.eGFP) into mice in which Cre was knocked into the Tac1 locus (Tac1^Cre^).^27^ Both methods revealed that the substance P-expressing neurons were concentrated in lamina IIo, and that there was no overlap with expression of PKCγ (Figure 4).

There is limited information about the relation between neuropeptide expression and morphology for these populations of excitatory interneurons. The GRP cells are morphologically diverse, and although a small number have vertical cell morphology,^84^ these are extremely rare (AJT and AM Bell, unpublished observations). There have apparently been no morphological studies of either the neurotensin or NKB cells, but these overlap extensively with PKCγ cells in lamina IIi-III,^53^ and again these are diverse in terms of their morphology.^102^ Although the substance P cells often have ventrally directed primary dendrites,^27^ our initial observations following intraspinal injection of AAVs coding for membrane-targeted fluorescent proteins^116^ in Tac1^Cre^ mice suggest that these cells are not vertical cells. For example, they do not have dendrites that extend ventrally into lamina III (AJT and E Polgár, unpublished data). It is therefore likely that excitatory interneurons expressing each of these four neuropeptides correspond to the central, radial or unclassified cells of Grudt and Perl.^38^ Interestingly, when mice in which Cre has been knocked into the prodynorphin locus (pdyn^Cre^) were crossed with a reporter line in which Cre drives tdTomato (tdTom) expression, some vertical cells were labelled with tdTom.^33^ Although dynorphin is expressed mainly by inhibitory interneurons, between 15% and 20% of dynorphin cells in laminae I-II are excitatory,^33,81^ and these presumably include the tdTom-positive vertical cells. It is therefore likely that although vertical cells are not included among the substance P, GRP, NKB or neurotensin populations, at least some of them express dynorphin.

Somatostatin is widely expressed among excitatory neurons in laminae I-II, although it is also present in inhibitory neurons in deeper dorsal horn laminae.^33,78^ We have estimated that somatostatin-immunoreactivity can be detected in ∼60% of the excitatory interneurons in laminae I-II,^53^ including the majority of those belonging to the substance P, GRP, NKB and neurotensin populations (Figure 3). This indicates that unlike these four neuropeptides, somatostatin is not restricted to a specific population among the excitatory interneurons, and this is consistent with the finding that somatostatin-expressing cells are morphologically and electrophysiologically heterogeneous.^33^ In addition, we have identified somatostatin in the axons of some (but not all) vertical cells in the rat.^19^ Vertical cells therefore appear to represent a discrete functional population among the excitatory interneurons, with some of these cells expressing dynorphin and/or somatostatin. Among the remaining excitatory interneurons (those with radial, central or unclassified morphology), expression of substance P, GRP, NKB and neurotensin defines four largely non-overlapping populations that are concentrated in specific sublaminae. However, it is not yet clear how they relate to these morphologically defined classes. It is also not known whether the subsets of PKCγ cells that express neurotensin or NKB represent different functional populations, or whether they correspond to a single class, with some expressing one or other peptide in an apparently random fashion.

The neurochemical populations described above account for over half of the inhibitory and excitatory interneurons in laminae I-II, but relatively few of those in lamina III. Two recent studies have provided further insight into the organisation of lamina III interneurons. Peirs et al.^34^ reported that transient expression of the vesicular glutamate transporter VGLUT3 defined a large population of excitatory interneurons in lamina III. These overlapped partially with the PKCγ cells and typically had dendrites that extended dorsal to the soma. Cui et al.^55^ identified a population of inhibitory interneurons, based on early expression of the receptor tyrosine kinase RET, that were mainly restricted to lamina III. These cells, which were morphologically heterogeneous, accounted for ∼one third of the inhibitory interneurons in this region, and overlapped with the parvalbumin, but not the dynorphin or nNOS, populations.

Taken together, the findings discussed above indicate that a neurochemical approach is likely to be useful for identifying functional populations among both the inhibitory and excitatory interneurons. However, considerable care is needed in defining populations, and it is likely that in most cases at least two different neurochemical markers will be required for this. An additional problem is that some of these markers, particularly the neuropeptides, may be transiently expressed in a wider group of cells. For example, we have found that when Tac1^Cre^ mice were crossed with a tdTom reporter line, many of the PKCγ-immunoreactive neurons in lamina IIi were tdTom^+^, even though these cells did not express eGFP^+^ following intraspinal injection of AAV.flex.eGFP^27^ (Figure 4). This suggests that many of the PKCγ cells transiently express substance P, but that this is switched off during development. Using a similar approach, we have also found evidence for transient expression of dynorphin in some inhibitory interneurons that do not express the peptide in the adult, including nNOS cells (AJT, M Gutierrez-Mecinas and E Polgár, unpublished data), and this is consistent with the finding that preprodynorphin mRNA could only be detected in ∼75% of tdTom neurons when pdyn^Cre^ mice were crossed with a tdTom reporter line.^33^

## Synaptic circuits involving interneuron populations

**Figure 5.**
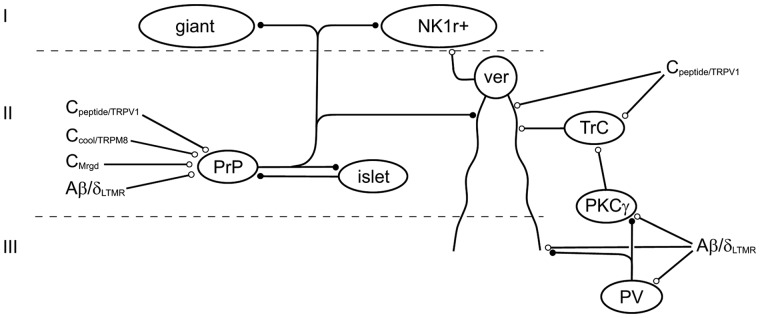
A schematic diagram showing some of the synaptic circuits discussed. Cells that express GFP under control of the prion promoter (PrP) correspond to a subset of inhibitory interneurons that express nNOS and/or galanin and dynorphin. These cells receive synaptic input from a variety of primary afferents, including C fibres that express TRPV1 or TRPM8, non-peptidergic C nociceptors that express MrgD and myelinated low-threshold myelinated afferents (A-LTMRs) that conduct in the Aδ or Aβ range. There is evidence that different classes of primary afferent converge on the same cell. They form reciprocal (inhibitory) synaptic connections with lamina II islet cells, and they are also presynaptic to lamina II vertical (ver) cells and to projection neurons in lamina I, which include giant cells. Lamina II vertical cells are thought to form part of a polysynaptic pathway that can transmit input from A-LTMRs to NK1r-expressing lamina I projection neurons. This pathway involves PKCγ-expressing excitatory interneurons in lamina IIi/III, together with transient central (TrC) cells in lamina II. A feedforward circuit involving inhibitory interneurons (including some that express parvalbumin, PV) normally limits the activation of PKCγ cells by A-LTMRs, and this could occur through both GABAergic presynaptic inhibition of the A-LTMR terminals and glycinergic postsynaptic inhibition of the PKCγ cells. A-LTMR afferents are also thought to innervate the ventral dendrites of vertical cells and this synaptic input may also be presynaptically inhibited by the PV cells. There is also evidence that both TrC and vertical cells receive nociceptive input from TRPV1-expressing primary afferents, indicating that the pathway involving these cells normally transmits nociceptive information. Note that dendrites are only illustrated on vertical cells to show that these enter lamina III, where they may receive A-LTMR input. Excitatory and inhibitory synapses are represented by open and closed circles, respectively. For further details, see text.

Numerous studies have investigated synaptic circuits that involve dorsal horn interneurons. A potential difficulty with interpretation of their findings is that, as noted above, some of the classes of interneuron that have been defined by morphology or by the expression of particular neurochemical markers may include more than one functional population, and these could have differing synaptic inputs and outputs. This section will therefore focus on studies that involve relatively restricted populations among the inhibitory or excitatory interneurons (Figure 5).

Among the inhibitory interneurons, those that express parvalbumin have been shown to give rise to axoaxonic synapses on the central terminals of A-LTMR afferents, and also to receive direct synaptic input from these afferents.^60^ The majority of parvalbumin-containing boutons in lamina IIi were presynaptic to these primary afferent terminals (identified by their glomerular synaptic arrangement^117^), although only around half of the A-LTMR terminals were associated with a parvalbumin-immunoreactive bouton. This suggests that the parvalbumin cells are an important source of presynaptic inhibition of myelinated low-threshold afferents, although other interneurons are also presumably involved in this form of inhibition. It has also been reported that parvalbumin axons form synapses on PKCγ neurons in lamina IIi.^61^ Consistent with the laminar location of the parvalbumin cells, we found that they did not upregulate the transcription factor Fos or phosphorylate extracellular-signal regulated kinases (ERK) following various types of noxious stimulation.^54^ It is therefore likely that their main input is from A-LTMRs^60^ and that they respond to tactile stimuli.

PrP-eGFP cells (which include some of the galanin/dynorphin- and nNOS-expressing inhibitory interneurons) can receive monosynaptic input from both unmyelinated and myelinated primary afferents.^56,57^ These include C fibres that express the transient receptor potential (TRP) channels TRPV1 or TRPM8, as well as A-LTMRs, and there is evidence that different types of primary afferent input converge onto individual cells.^49,56^ Kardon et al.^29^ recorded from a population of superficial dorsal horn neurons identified by expression of the transcription factor Bhlhb5. All of these were hyperpolarised by somatostatin, and were therefore presumably inhibitory interneurons belonging to the galanin/dynorphin or nNOS populations.^25,54,59^ Most of these cells showed evidence of monosynaptic input from TRPV1-, TRPA1- and TRPM8-expressing primary afferents. Duan et al.^33^ investigated cells in which tdTom expression was driven from the pdyn locus and reported that the vast majority of these cells were activated either mono- or polysynaptically following stimulation of Aβ afferents (most of which are LTMRs). Although the tdTom cells will have included some excitatory interneurons, as well as cells that transiently expressed dynorphin, this suggests that most dynorphin-expressing inhibitory interneurons receive A-LTMR input either directly or indirectly. Inhibitory interneurons in the rat that express galanin or nNOS have been shown to respond to a variety of noxious stimuli, based on expression of Fos or phospho-ERK (pERK).^54^ The galanin cells were frequently activated by noxious thermal and mechanical stimuli, as well as by chemical stimuli (subcutaneous capsaicin or formalin), whereas the nNOS cells were activated by noxious heat and subcutaneous formalin, but seldom by capsaicin. Electrophysiological studies have shown that the PrP-eGFP cells can be presynaptic to both islet and vertical cells in lamina II.^49^ They also receive inhibitory synaptic input from islet cells, and in some cases form reciprocal synapses with these cells. We found that although the axons of the PrP-eGFP cells arborise in lamina II, they often extend into laminae I and III. Their postsynaptic targets included NK1r-expressing neurons in this lamina (at least some of which are likely to be ALT projection cells). In addition, they targeted a specific type of lamina I projection neuron known as giant cells,^118,119^ which are characterised by a very high density of excitatory and inhibitory synaptic input. The giant cells received numerous synapses from PrP-eGFP axons that were nNOS-immunoreactive, and these accounted for ∼70% of their inhibitory synaptic input.^56^ However, all of the PrP-eGFP cells with axon entering lamina I also had extensive axonal arbors in lamina II. Taken together, these findings indicate that both the nNOS and galanin/dynorphin populations are likely to receive input from several different classes of primary afferent, including thermoreceptors, nociceptors and A-LTMRs, with many cells receiving convergent inputs from different types of afferent. However, they are likely to differ in their inputs, since the galanin/dynorphin cells were often activated by capsaicin, whereas the nNOS cells were not. Their postsynaptic targets include both interneurons and projection neurons, and although some of the nNOS cells innervate lamina I giant cells (providing their major inhibitory input), the giant cells probably represent only a minority of their postsynaptic targets.

We have recently performed a combined electrophysiological and morphological study of NPY-expressing cells in a NPY-eGFP mouse line.^58^ Although most eGFP^+^ cells in these mice were NPY-immunoreactive, they only accounted for ∼one third of the NPY-immunoreactive neurons in laminae I-II. Dorsal root stimulation revealed that the cells could receive monosynaptic input from C fibres, but they seldom responded to bath-applied capsaicin, and never to icilin, which suggests that few of them are innervated by TRPV1- or TRPM8-expressing primary afferents. However, it is possible that NPY cells in laminae I-II that lacked eGFP in this mouse line receive inputs of this type. For four of the recorded cells (three of which had cell bodies in lamina III), we found that monosynaptic C fibre-evoked excitatory postsynaptic currents (EPSCs) were resistant to bath-applied capsaicin, indicating that they were from TRPV1-negative afferents. The recordings were made in the medial region of the lumbar enlargement, which receives input from glabrous skin and therefore lacks C-LTMRs. It is therefore likely that these inputs originated from C^MrgD^ nociceptors, most of which lack TRPV1,^7^ and which are thought to be involved in mechanical pain.^8^ Consistent with this interpretation, we found that lamina III NPY cells often showed pERK following pinching of the skin. We have also reported that in the rat many NPY cells in laminae I-II are activated by mechanical, thermal and chemical noxious stimuli.^54^ Postsynaptic targets of the NPY cells have been identified in the rat, and include ALT projection neurons in lamina III as well as PKCγ interneurons in lamina IIi,^120,121^ with both of these populations receiving around 30% of their inhibitory synapses from GABAergic boutons that contain NPY. Lamina III ALT neurons in the mouse are also innervated by NPY cells,^15^ and this input appears to originate from a specific subset of the NPY cells.^58^ However, for those NPY cells that innervate lamina III ALT neurons, this seems to represent only a minority of their axonal output.

Relatively little is known about the synaptic circuitry involving excitatory interneuron populations. Several studies have investigated vertical cells in lamina II, and these have been shown to receive monosynaptic excitatory input from both C and Aδ afferents.^38,40,46,49^ Since their dendrites often extend at least as far as lamina IIi, the Aδ input could originate from either nociceptive or LTMR afferents, and it is not known whether both of these types directly innervate the cells. We have found that the ventral dendrites of vertical cells are frequently contacted by axons that contain the vesicular glutamate transporter VGLUT1,^122^ which would be consistent with a direct synaptic input from A-LTMRs (including Aδ D-hair afferents).^6,79^ There is disagreement about the extent to which vertical cells are innervated by primary afferents that express TRP channels. Zheng et al.^49^ found no increase in the frequency of miniature EPSCs in the presence of either capsaicin or icilin, which would suggest that vertical cells receive little or no input from TRPV1- or TRPM8-expressing primary afferents. However, most of the vertical cells recorded by Uta et al.^123^ showed an increased mEPSC frequency in response to both capsaicin and cinnamaldehyde, suggesting monosynaptic input from afferents expressing TRPV1 and TRPA1. Since non-peptidergic C nociceptors (which correspond to the C^MrgD^ population in mouse) express TRPV1 in rats, but not in adult mice,^124^ it is possible that the vertical cells are innervated by these, but not by peptidergic afferents (which continue to express TRPV1 in both species). Dorsal root stimulation can evoke inhibitory postsynaptic currents (IPSCs) in vertical cells at latencies consistent with activation of both Aδ and C fibres, indicating that inhibitory interneurons that synapse on these cells are activated by both myelinated and unmyelinated afferents.^40^ At least some of this inhibitory input is likely to originate from PrP-eGFP cells (i.e., those belonging to the nNOS and/or galanin/dynorphin populations), since Zheng found that these cells (but not islet cells) provided direct synaptic input to vertical cells.^49^ Lu and Perl^46^ also identified direct synaptic inputs to vertical cells from another population of excitatory interneurons that they defined as “transient central” cells. Based on paired recordings, Lu and Perl^46^ were also able to identify lamina I neurons as postsynaptic targets of vertical cells, and some of these lamina I neurons were shown to have long ascending axons and to respond to bath-applied substance P. This strongly suggests that lamina I projection neurons that express the NK1r receive excitatory synaptic input from lamina II vertical cells. Consistent with this interpretation, trans-synaptic retrograde labelling from the lateral parabrachial area (the major target for lamina I ALT neurons) revealed a population of lamina II vertical cells that were presumably presynaptic to the ALT neurons in lamina I.^125^ However, vertical cells also have extensive axonal arborisation in lamina II (and sometimes in deeper laminae), while most lamina I ALT neurons have dendrites that remain within this lamina. This indicates that lamina I projection neurons represent only a minority of the synaptic output from vertical cells.

As stated above, PKCγ-expressing excitatory interneurons in lamina IIi-III have been implicated in mechanical allodynia in chronic pain states.^100,103–105^ Torsney and MacDermott^126^ showed that large lamina I NK1r-expressing neurons (which are likely to have been projection cells) normally receive minimal input from Aβ afferents. However, when synaptic inhibition in the dorsal horn was blocked, these cells showed polysynaptic Aβ inputs. This was thought to represent a substrate for mechanical allodynia, with output neurons that were normally driven by noxious stimuli acquiring low-threshold tactile inputs. The PKCγ cells have been shown to receive synapses from VGLUT1-containing axons,^127^ which presumably include A-LTMRs. In an elegant paired-recording study in the rat, Lu et al.^105^ showed that they also receive an inhibitory input from glycinergic interneurons in lamina III, which presumably include the PV cells.^61^ Both the PKCγ cell and the lamina III inhibitory interneuron were innervated by Aβ afferents, forming a feed-forward inhibitory circuit that could suppress low-threshold mechanoreceptive input to the PKCγ cells.^105^ Lu and Perl^46^ also demonstrated that PKCγ cells formed excitatory synapses onto the transient central cells in lamina II, and these have been shown to target vertical cells, which in turn can synapse on lamina I projection neurons. The circuit proposed by Lu et al.^105^ (see Figure 5) would therefore provide a polysynaptic route through which tactile input from A-LTMRs could gain access to lamina I projection neurons, consistent with the findings of Torsney and MacDermott.^126^ Interestingly, following ligation of the L5 spinal nerve,^128^ the glycinergic inhibition of PKCγ cells was reduced, while the excitatory input from PKCγ to transient central cells was strengthened.^105^ Stimulation of the L5 dorsal root at Aβ strength now resulted in polysynaptic EPSPs and action potential firing in transient central cells, vertical cells and NK1r-expressing lamina I neurons in this segment. However, these changes were apparently not seen when the intact L4 dorsal root was stimulated. Since the input from intact L4 afferents is thought to underlie mechanical allodynia in this model,^129,130^ it is not clear to what extent the changes observed after nerve injury contribute to this type of allodynia.

Duan et al.^33^ recorded from somatostatin-expressing interneurons in lamina II and found that most of these were innervated by C fibres, with some also receiving input from Aδ and/or Aβ afferents. Subsequent analysis revealed that these neurons were morphologically heterogeneous, consistent with the widespread expression of somatostatin among various excitatory interneuron populations.

One further example of selective circuitry involving excitatory interneurons involves lamina III ALT projection neurons. These cells receive numerous excitatory synapses from peptidergic primary afferents,^131^ but they are also innervated by non-primary glutamatergic boutons, which can be identified by expression of VGLUT2 and are likely to originate from local excitatory interneurons.^132^ We found that in the rat ∼60% of VGLUT2 terminals contacting these cells were immunoreactive for preprodynorphin, and that these formed synapses on the dendrites of the ALT neurons. Since preprodynorphin was only seen in ∼5% of VGLUT2 boutons in laminae I-IV, this indicates a highly selective innervation of the lamina III ALT neurons by dynorphin-containing excitatory interneurons.^81^ However, it is not known whether these interneurons have their cell bodies in the superficial or deep dorsal horn.

## Neuropeptide signalling

Although the functions of dorsal horn interneurons are presumably mediated mainly through the synaptic action of the fast transmitters glutamate, GABA and glycine, the presence of numerous peptides and the corresponding receptors in this region indicates that neuropeptide signalling is also likely to play a significant role in somatosensory processing.

Substance P released from nociceptive primary afferents^133,134^ and excitatory interneurons^27^ will act on NK1 receptors, which are expressed mainly by ALT projection neurons and excitatory interneurons in lamina I.^15,135^ The resulting depolarisation of these cells presumably contributes to the perception of pain because mice lacking substance P or the NK1 receptor show reductions in pain behaviour.^136,137^ However, the majority of substance P in the dorsal horn is in primary afferent terminals, with only ∼20% originating from local neurons,^138^ and it is therefore not clear to what extent substance P released from local interneurons contributes to this effect.

Like substance P, somatostatin will also be released from both primary afferents and axons of local excitatory interneurons, but in this case the excitatory interneuron terminals are likely to be much more numerous than those of primary afferent origin.^139^ The main receptor for somatostatin in the dorsal horn is sst_2A_, which is expressed on some primary afferent terminals and on a subset of inhibitory interneurons that include the galanin/dynorphin and nNOS populations.^54,89,140^ Since somatostatin has a hyperpolarising (inhibitory) action on dorsal horn neurons,^19,59,141^ the overall effect will presumably be disinhibition. Intrathecal injection of somatostatin, or its analogue octreotide, causes scratching, biting and licking behaviour that are thought to result from itch.^29,142^ In addition, a subthreshold intrathecal dose of octreotide increased the duration of biting in response to intradermal injection of the pruritogen chloroquine but had no effect on the latency to withdraw the hindlimb on a hotplate.^29^ Interestingly, intrathecal octreotide caused very little scratching or biting in *Bhlhb5^-/-^* mice, which lack the galanin/dynorphin and nNOS inhibitory interneurons. These findings suggest that somatostatin released by primary afferents and/or local excitatory interneurons acts by suppressing activity in neurons that normally inhibit the spinal itch pathway.^29^

There has been considerable debate about the sources of GRP in the spinal cord. Although early studies reported that it was expressed by primary afferents,^93^ several groups have failed to detect mRNA for GRP in dorsal root ganglion^115,143,144^ and it is now thought that the GRP-like immunoreactivity seen in primary afferents results from cross-reaction with substance P.^84,115^ Several lines of evidence point to a role for GRP and the GRPR in certain types of chemical itch^93,94^: (1) mice lacking the GRPR show significantly reduced itch, particularly in response to non-histaminergic pruritogens such as chloroquine; (2) intrathecal GRPR agonists evoke scratching, while antagonists reduce scratching in response to injected pruritogens; and (3) ablation of spinal GRPR-expressing neurons with saporin conjugated to bombesin (an amphibian homologue of GRP) results in reduced responsiveness to pruritogens. Since excitatory interneurons in lamina II appear to be the main source for spinal GRP, this would suggest that they are among those activated by pruritogens such as chloroquine. However, we have found that the GRP cells have a relatively low probability of expressing Fos or pERK following intradermal injection of chloroquine.^145^ Their involvement in spinal itch mechanisms will therefore require further investigation.

NKB or neurotensin can be released by some excitatory interneurons in lamina IIi-III. NKB acts on the neurokinin 3 receptor (NK3r), which is expressed by at least two populations of superficial dorsal horn interneurons:^146,147^ inhibitory interneurons that contain nNOS and a population of excitatory interneurons that co-express the μ-opioid receptor.^148^ However, at present, little seems to be known about the role of NKB and the NK3r in spinal somatosensory processing. Although receptors for neurotensin have been identified in the spinal cord, there is apparently no information on the types of interneuron on which these are expressed.

NPY released from inhibitory interneurons can act on the NPY Y1 receptor, which is expressed by many dorsal horn neurons, including both projection cells and excitatory interneurons in the superficial laminae.^90,92^ Since activation of this receptor causes hyperpolarisation,^149^ this is likely to contribute to the anti-nociceptive actions of NPY.^150^ However, interpretation of the behavioural effects of NPY is complicated by the presence of functional Y1 receptors on nociceptive primary afferent terminals.^151^ Interestingly, knockdown of NPY from the spinal cord did not affect baseline pain thresholds, but caused the reappearance of behavioural signs of neuropathic pain after these had resolved in nerve-injured animals.^152^ This suggests that NPY signalling is more important in pathological pain states than in normal sensation. Galanin is contained in a different population of inhibitory interneurons^109^ and can act on galanin1 receptors, which are expressed by many excitatory interneurons in the superficial laminae.^153,154^ As with NPY, release of galanin may therefore inhibit excitatory interneurons, leading to a reduction in nociceptive transmission in the dorsal horn, but again the situation is complicated by the presence of galanin receptors on primary afferent terminals.^150^

Dynorphin acts on the κ opioid receptor (KOR), and although relatively little is known about the spinal distribution of KORs, this signalling pathway has been implicated in itch.^29^ Intrathecal delivery of two different KOR agonists to the lumbar spinal cord suppressed itch evoked by intradermal injection of chloroquine in the calf, whereas administration of KOR antagonist led to an increase in chloroquine-evoked biting.^29^ KOR agonists also reduced the itching caused by intrathecal administration of GRP, suggesting that the KOR action was downstream of the GRP-GRPR mechanism. Direct evidence that dynorphin released from spinal interneurons is involved in suppressing itch was provided by showing that chloroquine-evoked itch could be inhibited by menthol in wild-type mice, but not in *Bhlhb5^−/−^* mice, in which the dynorphin cells are largely absent.^29^

## What we know about the functions of different interneuron populations

Several recent studies have investigated the roles of populations of inhibitory or excitatory interneurons in the dorsal horn in vivo by targeting them with molecular genetic strategies. Mice in which recombinases (generally Cre) were expressed in specific populations were either crossed with other mouse lines that expressed Cre-dependent proteins or received intraspinal injections of viruses coding for these proteins. This then allowed a variety of manipulations, including ablation or silencing of neurons with toxins, as well as activation or inactivation of the cells, achieved through either chemogenetic or optogenetic approaches.

Foster et al.^28^ demonstrated an important role for glycinergic neurons, by injecting AAVs coding for Cre-dependent toxins into the spinal cords of mice in which Cre was expressed under control of the GlyT2 promoter. Glycinergic cells account for a large proportion (∼90%) of the inhibitory interneurons in deeper dorsal horn (laminae III-VI) and ventral horn, but only around 20% of those in lamina I-II. However, they include most of the nNOS cells in the superficial laminae.^28,108^ Ablation of the GlyT2-expressing neurons with diphtheria toxin A fragment (DTA) or synaptic silencing with tetanus toxin light chain resulted in hypersensitivity to both thermal and mechanical stimuli, as well as spontaneous aversive behaviour. The mice also showed excessive scratching and biting, resulting in hair loss in the affected dermatomes, and this was thought to reflect spontaneous itching. Chemogenetic activation of the glycinergic neurons by means of clozapine-N-oxide (CNO) acting on the modified muscarinic receptor hM3Dq^155^ increased the threshold for mechanical and thermal noxious stimuli, reduced signs of neuropathic pain following peripheral nerve injury and also reduced responses to pruritic stimuli (intradermal injection of chloroquine and histamine).

Inhibitory interneurons that express parvalbumin are included among the glycinergic neurons in laminae IIi-III, although there is also a population of parvalbumin-expressing excitatory interneurons in this region.^95,96^ Petitjean et al. showed that activation of parvalbumin cells, mediated through the action of CNO on hM3Dq, increased withdrawal thresholds to mechanical (but not thermal stimuli) and attenuated mechanical allodynia following peripheral nerve injury. They also showed that ablation of the cells with saporin led to mechanical allodynia, but no change in responses to thermal stimuli.^61^ The suppression of mechanical allodynia appeared to involve PKCγ-expressing excitatory interneurons,^61^ suggesting that parvalbumin cells may correspond (at least in part) to the glycinergic lamina III neurons that provide feedforward inhibition of A-LTMR input to the PKCγ cells^105^ (Figure 5).

Cui et al.^55^ tested the effects of ablating (with DTA) or activating (via hM3Dq) inhibitory interneurons that were defined by early expression of RET. They found that ablating these cells, which are mainly located in lamina III and include some of the parvalbumin cells, resulted in mechanical allodynia, together with enhanced responses to acute noxious (mechanical and thermal) stimuli and increased hyperalgesia in both inflammatory and neuropathic pain states. In contrast, activating them led to a reduction of acute pain and of both neuropathic and inflammatory hyperalgesia.

Mice lacking the transcription factor Bhlhb5 show exaggerated itch, which is thought to result from loss of inhibitory interneurons in the superficial dorsal horn.^113^ This loss of interneurons is restricted to the nNOS- and dynorphin/galanin-expressing cells, and it has been proposed that one or both of these populations of inhibitory interneurons contributes to the suppression of itch by counterstimuli.^29^ Dynorphin released by these cells can reduce itch through a spinal action on κ-opioid receptors, but is thought that a fast synaptic transmitter (GABA or glycine) underlies the immediate relief provided by counterstimuli, such as scratching.^29^ However, Duan et al.^33^ reported that although ablation of Cre-expressing spinal neurons in pdyn^Cre^ mice resulted in a dramatic increase in responses to noxious mechanical (but not thermal) stimuli, it had no effect on itch behaviour following intradermal injection of various pruritogens. As noted above, there is transient expression of dynorphin in some dorsal horn interneurons, and the population ablated in this study may therefore have included these cells.

NPY-expressing cells have been targeted in a mouse line in which Cre expression is under control of the NPY promoter.^33,67^ However, it was reported that when this line was crossed with a reporter strain to induce tdTom in Cre-containing neurons, only a third of the tdTom^+^ cells in adult mice expressed NPY, presumably because of transient expression of NPY by a much larger population of inhibitory interneurons during development. Ablation of the Cre-expressing cells had no effect on mechanical or thermal nociceptive thresholds or on the responses to injected pruritogens. However, ablation or silencing of these neurons did result in spontaneous scratching of hairy skin that led to skin lesions, and this was thought to reflect loss of inhibition of mechanical itch.

Taken together, the results of these studies suggest that inhibitory interneurons in the deep dorsal horn (laminae III-VI) are involved in suppressing all types of acute and pathological pain.^28,55,61^ Among these cells, those that express parvalbumin appear to be particularly important for preventing mechanical allodynia,^61^ and this is consistent with their proposed role in the presynaptic inhibition of A-LTMRs.^60^ In contrast, the dynorphin-expressing inhibitory interneurons (which are restricted to the superficial laminae) can suppress mechanical, but not thermal, pain. The situation with itch is more complex: The itching evoked by intradermal injection of pruritogens, such as chloroquine and histamine, is increased in *Bhlhb5^−/−^* mice, suggesting an antipruritic role for the dynorphin/galanin and/or nNOS populations.^29^ However, ablation of Cre-expressing inhibitory interneurons in the pdyn^Cre^ mouse (which may also include nNOS cells) had no significant effect on chloroquine-evoked itch.^33^ In addition, the GlyT2 population, which includes many of the nNOS cells in superficial dorsal horn, seems to be involved in suppressing itch.^28^ It will therefore be of interest to determine whether selective activation of either dynorphin/galanin or nNOS populations can reduce the itching caused by pruritogens. Finally, a population of cells labelled in a NPY-Cre line can suppress mechanical itch from hairy skin. However, this is not likely to be the only role of the NPY cells because the plexus of NPY-containing axons that originates from these cells is present throughout the mediolateral extent of the dorsal horn in mid-lumbar regions,^120^ including the medial part, which corresponds to glabrous skin territory.

A number of studies have investigated the roles of excitatory interneuron populations. Duan et al.^33^ showed that ablation of somatostatin-expressing neurons led to a dramatic reduction in mechanical pain, as well as absence of mechanical allodynia in both inflammatory and neuropathic pain states. This loss of allodynia was thought to result from disruption of transmission from Aβ LTMRs through circuits in the superficial dorsal horn. Consistent with these findings, Christensen et al.^68^ reported that optogenetic activation of somatostatin interneurons resulted in a nocifensive response, involving licking of the appropriate dermatome. Tests for conditioned place preference confirmed that this was an aversive stimulus. They also demonstrated that chemogenetic inhibition of somatostatin cells increased acute mechanical withdrawal thresholds and reduced mechanical allodynia in an inflammatory model. However, unlike Duan et al., they also saw an effect on noxious thermal stimuli, with a slight increase in withdrawal latency following chemogenetic inhibition of the somatostatin cells.

Duan et al.^33^ also ablated two additional populations among the excitatory neurons: those expressing NKB or calretinin. Loss of the NKB cells had no effect on mechanical pain, while loss of the calretinin cells resulted in a significant increase in the withdrawal threshold for von Frey hairs, but no change in repsonses to pinch or pinprick and no signs of mechanical allodynia. Since calretinin is expressed in a large number of excitatory interneurons, the relatively modest change seen after ablation of these cells is surprising. However, around 12% of calretinin cells in the superficial dorsal horn are inhibitory,^62^ and it is possible that ablation of calretinin-expressing inhibitory interneurons partially compensated for the reduction of nociceptive transmission due to loss of the excitatory calretinin cells. The findings described above clearly implicate excitatory interneurons in laminae I-II in pathways responsible for mechanical pain. However, the somatostatin-expressing cells seem to be a fairly heterogeneous population,^33,53^ and it will be important to determine the roles of more restricted populations, such as vertical cells, or excitatory interneurons that express GRP or substance P.

Mice lacking VGLUT3 show attenuated acute mechanical pain and reduced mechanical allodynia after nerve injury or inflammation.^10^ Peirs et al.^34^ recently demonstrated that this can be replicated by deletion of VGLUT3 from the “transient VGLUT3” population of excitatory interneurons that are mainly restricted to lamina III, thus implicating these cells in mechanical pain and hypersensitivity. They provided further evidence for this interpretation by chemogenetically activating these cells via hM3Dq, and showing that this resulted in mechanical allodynia with no change in response to noxious thermal stimuli. In addition, they activated calretinin neurons in adult mice, and demonstrated that this also caused mechanical allodynia, suggesting that calretinin-expressing excitatory interneurons in the superficial dorsal horn play a significant role in transmitting noxious mechanical information.

## Conclusions

The findings discussed above indicate that the neuronal organisation of the dorsal horn is highly complex, and we still do not have a comprehensive scheme for classifying the excitatory and inhibitory interneurons. This has inevitably limited our understanding of the synaptic circuits that transmit and modulate somatosensory information within the dorsal horn. It is clear that inhibitory interneurons can suppress pain and itch, while excitatory interneurons contribute to both acute and pathological pain, but it is likely that different populations have relatively specific roles within these broadly defined functions. The identification of specific molecular/genetic markers to allow targeting of interneuron populations, together with dramatic advances in the development of methods for manipulating neuronal function in vivo, have already provided major breakthroughs in our understanding of somatosensory processing. However, care is needed when selecting these genetic markers because in many cases, they are likely to be expressed in more than one functional population, either permanently or transiently. It is therefore likely that intersectional genetic approaches, probably requiring the use of two different recombinases,^33^ will be required.

Many important questions remain to be addressed. For example, which types of inhibitory interneuron are responsible for presynaptic inhibition of primary afferents, including the C^MrgD^ nociceptors, which are known to receive axoaxonic synapses.^117,156^ Several independent studies have proposed a circuit for mechanical allodynia that involves three different types of excitatory interneuron: PKCγ cells, transient central cells and vertical cells (Figure 5). It will be important to determine whether any of the neurochemical markers so far identified correspond to the transient central cells, and also to find a way of selectively manipulating the functions of vertical cells. A more general question is whether the non-overlapping neurochemical subsets that have been identified among both inhibitory^54^ and excitatory^27^ interneurons correspond to genuine functional populations. To answer this, it will be necessary to compare other parameters, such as firing patterns and synaptic inputs, within and between these groups. Further morphological studies will be needed to test whether the radial cells identified by Grudt and Perl^38^ have consistent neurochemical properties or synaptic relationships and thus represent a distinct population. Little attention has so far been paid to long intraspinal connections, and it will therefore be of interest to see which populations of interneurons project to rostral and/or caudal spinal segments.
